# Expression of Claudin-9 (CLDN9) in Breast Cancer, the Clinical Significance in Connection with Its Subcoat Anchorage Proteins ZO-1 and ZO-3 and Impact on Drug Resistance

**DOI:** 10.3390/biomedicines11123136

**Published:** 2023-11-24

**Authors:** Xinguo Zhuang, Tracey A. Martin, Fiona Ruge, Jianyuan (Jimmy) Zeng, Xinyu (Amber) Li, Elyas Khan, Qingping Dou, Eleri Davies, Wen G. Jiang

**Affiliations:** 1 School of Medicine, Cardiff University, Cardiff CF14 4XN, UK; zhuangx5@cardiff.ac.uk (X.Z.); martinta1@cardiff.ac.uk (T.A.M.); ruge@cardiff.ac.uk (F.R.); lix193@cardiff.ac.uk (X.L.); douq1@cardiff.ac.uk or doup@karmanos.org (Q.D.); 2 Department of Clinical Laboratory, The First Affiliated Hospital of Xiamen University, School of Medicine, Xiamen University, Xiamen 361003, China; 3 Karmanos Cancer Institute, Department of Oncology, School of Medicine, Wayne State University, Detroit, MI 48201, USA; elyaskhan882@gmail.com; 4 Wales Breast Centre, University Llandough Hospital, Cardiff and Vales University Health Board, Cardiff CF64 2XX, UK; eleri.davies8@wales.nhs.uk

**Keywords:** claudin, CLDN9, ZO-1, ZO-3, breast cancer, prognosis, chemotherapies, chemoresistance, cytotoxicity, Her-2, neratinib, estrogen receptor

## Abstract

(1) Introduction: Claudin-9 (CLDN9) is a member of the claudin protein family, a critical transmembrane protein family for tight junctions that are implemented in the progression of numerous cancer types. The present study investigated the role that CLDN9, along with the subcoat proteins, Zonula Occludens (ZOs), plays in clinical breast cancer and subsequent impact on drug response of patients. (2) Methods: CLDN9 protein and *CLDN9* transcript were determined and correlated with clinical and pathological indicators, together with the status of hormonal receptors. The levels of *CLDN9* transcript were also assessed against the therapeutic responses of the patients to chemotherapies by using a dataset from the TCGA database. Breast cancer cell models, representing different molecular subtypes of breast cancer, with differential expression of CLDN9 were created and used to assess the biological impact and response to chemotherapeutic drugs. (3) Results: Breast cancer tissues expressed significantly higher levels of the *CLDN9*, with the high levels being associated with shorter survival. CLDN9 was significantly correlated with its anchorage proteins ZO-1 and ZO-3. Integrated expression of CLDN9, ZO-1 and ZO-3 formed a signature that was significantly linked to overall survival (OS) (*p* = 0.013) and relapse-free survival (RFS) (*p* = 0.024) in an independent matter. *CLDN9* transcript was significantly higher in patients who were resistant to chemotherapies (*p* < 0.000001). CLDN9 connection to chemoresistance was particularly prominent in patients of ER-positive (ER(+)), Her-2-negative((Her-2(−)), ER(+)/Her-2(−) and triple-negative breast cancers (TNBCs), but not in patients with HER-2-positive tumors. In Her-2-negative MCF7 and MDA-MB-231 cancer cells, loss of CLDN9 significantly increased sensitivity to several chemotherapeutic drugs including paclitaxel, gemcitabine and methotrexate, which was not seen in Her-2(+) SKBR3 cells. However, suppressing Her-2 using neratinib, a permanent Her-2 inhibitor, sensitized cellular response to these chemodrugs in cells with CLDN9 knockdown. (4) Conclusions: CLDN9 is an important prognostic indicator for patients with breast cancer and also a pivotal factor in assessing patient responses to chemotherapies. Her-2 is a negating factor for the treatment response prediction value by CLDN9 and negating Her-2 and CLDN9 may enhance breast cancer cellular response to chemotherapeutic drugs.

## 1. Introduction

Claudins (CLDNs) are a family of small transmembrane proteins that are key elements for tight junctions. This protein family has twenty-four members each sharing a similar protein structure that has four membrane-spanning domains with both the C- and N-terminus located in the cytoplasmic region. Intracellularly, the N-terminus anchors the claudin protein to the cytoskeleton via interaction with proteins such as the Zonula Occludens (ZOs). Both termini are also involved in mediating cell signaling. Claudins, by interacting with the same or different claudins on the other cells via the extracellular domains, form a key part of the control mechanism of paracellular permeability and hence the function of tight junctions. The role of claudins in pathological conditions particularly in cancers including breast cancer have been explored in recent years. For example, CLDN1, CLDN3, CLDN7, CLDN16 and CLDN20 have been found to be downregulated in breast cancer, and the downregulation of these claudins appearing to be linked to a more aggressive phenotype and with poor clinical outcome [1,2,3,4,5,6,7,8]. In contrast, CLDN3, CLDN4 and CLDN5 appear to be overexpressed in breast cancers [9,10,11,12,13]. However, determining whether claudins play a tumor-suppressive or oncogenic role is dependent on both the individual claudin and the type of cancer. Perhaps the most well-studied claudin in breast cancer is CLDN4, which has been found to be highly aberrant and has a role to define a subgroup of breast cancer [2,14,15,16,17]. As well as an indicator for disease progression and prognosis in various cancers, some claudins have also been shown to be indicators for cancer cell response to anticancer drugs. For example, CLDN4 overexpression, an indicator of poor clinical outcome in patients with ovarian cancer, is an indicator of resistance to cisplatin in this cancer type [18,19], and a similar role for CLDN6 is seen in cervical adenocarcinoma [20]. In breast cancer cells such as MCF-7, CLDN6 expression confers cellular resistance to drugs such as 5-fluouracil (5-FU) and adriamycin [21]. From a clinical point of view, high CLDN2 in colorectal cancer appears to be linked to poor outcome in those receiving 5-FU treatment [22] and CLDN1 and CLDN7 appear to form a claudin signature to predict the clinical response to chemotherapies in colorectal cancer [23]. The CLDN2 link may be via the paracellular passage of drugs in lung cancer cell models [24,25]. In breast cancer and particularly in triple-negative breast cancer (TNBC), CLDN1 is a good indicator for cancer cell response to paclitaxel, doxorubicin and 5-FU [26]. However, in colorectal cancer, CLDN1 often becomes upregulated following chemotherapies, and overexpression of CLDN1 confers cells’ resistance to oxaliplatin [27].

Compared with other members, CLDN9 is one of less well-studied claudins. CLDN9 protein is encoded by the *CLDN9* gene located on chromosome 16p13.3 and is 217 amino acids in size (22.8 kDa). It was first reported as a gene product similar to a gene in rats called Rat Ventral Protein.1 (RVP.1) [28,29]. Mouse studies revealed that this gene is required for the preservation of sensory cells in ears and the gene deficiency is associated with deafness [30,31].

CLDN9 is known to be a key binding protein for pathogens. It is a coreceptor to hepatitis C virus [32,33], and is also a receptor for the *Clostridium perfringens* enterotoxin (CpE) in the gut [34]. In gastric cancer, strong CLDN9 protein staining in the tissues of diffuse type is associated with high mortality [35], a finding seemingly replicable in a cell line of gastric cancer [36]. CLDN9 is generally well expressed in the pituitary gland and pituitary oncocytomas and it was seen to be highly linked to the invasive subtype of the tumors [37,38]. In cervical cancers, CLDN9, amongst other claudins, is seen to associate with lymphatic invasion [39] and is one of the few genes in endometrial cancer to predict a survival of the patients [40,41]. CLDN9 was found to be linked with metastasis of in vivo lung cancer models and with cell migration and invasiveness in vitro [42].

Studies on CLDN9 in breast cancer is rather rare. It has been reported to be low or negative along with CDLN6, CLDN12 and CLDN13 in breast tissues [43]. We have previously studied the role of claudins and tight junction molecules including CLDN19, CLDN20, and ZOs in breast cancer [6,7,44]. In the present study, we identified CLDN9 as potentially an important factor in assessing the clinical outcome of the patients and also patient response to drug treatment, and that this connection appears to be hormone receptor-dependent. We further developed cell models expressing differential CLDN9 expression levels and different hormonal receptor status, to confirm that in Her-2-positive breast cancer cells (SKBR3), knockdown of CLDN9 rendered the cells more sensitive to chemodrugs when Her-2 inhibitor is present. Collectively, CLDN9 is a prognostic indicator for breast cancer and a predictor for patient therapeutic response in Her-2-negative breast cancers.

## 2. Materials and Methods

### 2.1. Cell Lines

The following human breast cancer cell lines, MCF7, MDA-MB-231 and SKBR3, were purchased from ATCC (American Type of Cell culture) (purchased via LGC Standards, Teddinton, England, UK) and cultured in Dubecco’s Modified Eagle Medium (DMEM) with 10% foetal calf serum (FCS) (Sigma-Aldrich, Dorset, UK) and 1 × antimicrobial solutions (Sigma-Aldrich, Dorset, England, UK).

### 2.2. Mammary Tissue Cohort

Breast cancer tissue and background normal tissues (*n* = 127, and *n* = 33) were collected immediately after surgical removal of breast cancer at the University Hospital of Wales, as reported previously [45]. None of the patients received chemotherapy prior to surgery. Written informed consent was required and obtained from patients, with a follow-up study with a median follow-up period of 120 months conducted after the surgery. Pathological, clinical and follow-up information were obtained from clinical records and used for subgroup analyses. Of the all the patients, nine patients died of reasons unrelated to breast cancer and were excluded from the subgroup analysis. Sample tissue was sectioned using a cryostat (Leica CM1950) (Leica Biosystems Ltd., Newcastle, England, UK). The samples were collected under ethical approval (Bro Taf Health Authority; ethics approval numbers 01/4303 and 01/4046). Part of the frozen tissue sections were used for routine histological evaluation while the remaining sections were blended and homogenized before being subject to Tri Reagent RNA (Meck Sigma Aldrich, Dorset, UK) extraction for further genetic analysis.

### 2.3. Key Research Materials

A mouse monoclonal antibody to GAPDH(SC-32233), a goat polyclonal antibody to CLDN9(SC-17672) and a mouse monoclonal antibody to CLDN9(SC-398836) were, respectively, purchased from Santa-Cruz Biotechnologies Inc. (Santa Cruz, CA, USA). A mouse monoclonal antibody to ZO-1(33-9100) was, respectively, purchased from Thermo Fisher Scientific Inc. (Thermo Fisher Scientific, Loughborough, UK). siRNA targeting human CLDN9 was obtained from Santa-Cruz Biotechnologies Inc. Chemo-drugs, including gemcitabine (GEM), docetaxel (DOC), cisplatin (CIS), methotrexate (MTX) and docetaxel were purchased from Sigma-Aldrich (Dorset, UK). A broad-spectrum permanent Her-2 inhibitor, neratinib, was obtained from PUMA Biotechnologies Inc. (Los Angeles, CA, USA). These drugs were dissolved in DMSO, further diluted with DMEM to a desired concentration and stored at −20 °C until use. Other chemicals were purchased from Merck unless otherwise stated.

### 2.4. Creation of CLDN9 Knockdown Cell Models

Breast cancer cell lines MCF7, MDA-MB-231 and SKBR3 were used to create sublines with CLDN9 knockdown. Transient knockdown using siRNA (SC-43050) from Santa Cruz Biotechnology Inc., Dallas, TX, USA, was used on the cells. The sequence of siRNA for CLDN9 were as follows: Sense: GAGCAUUUGUAACUGGAAAtt. Antisense: UUUCCAGUUACAAAUGCUCtt. Transfection of cancer cells was carried out using transfection kits (SC-36868 and SC-29528) purchased from Santa Cruz Biotechnology Inc., by following the manufacturer’s instructions. The effect of knockdown was verified using PCR, qPCR and Western blotting analyses.

### 2.5. Analysis of Gene Transcript by PCR and QPCR

Quantitative and qualitative gene transcript analyses were undertaken using real-time quantitative RT-PCR and QPCR by employing the Amplifluor Molecular Beacon system. Reactions were prepared in a MicroAmp fast Optical 96-well plate (Greiner Bio-One Ltd., Gloucestershire, UK) using primers specific to the molecule of interest. In addition to unknown samples, reactions were prepared for a known standard that was run alongside the unknown samples. Once all samples and unknowns were added to the plate, the plate was sealed with optical seals (PrimerDesign, Southampton, UK) and the sample was subjected to a StepOne Plus qPCR system (Thermo Fisher Scientific, Waltham, MA, USA). Relative copy numbers of the samples were calculated as part of the systematic analysis, in accordance with the standard curve, and were subsequently exported to Excel 2019 (Microsoft Inc., Redmond, WT, USA) for further analysis. Qualitative PCR product was separated using 1% agarose and image obtained from a UV imager. Sequences used in the study were as follows: QPCR for *CLDN9*: 5′GTGCCCTCTGTGTCATTG′3 and 5′ACTGAACCTGACCGTACATCCACACACGTGGTACACT′3, ZO-1: 5′CCACATACAGATACGAGTCCTC′3 and 5′ACTGAACCTGACCGTACAGTAACTGCGTGAATATTGCT′3; ZO-2: 5′ CAAAAGAGGATTTGGAATTG′3 and 5′ ACTGAACCTGACCGTACAGAGCACATCAGAAATGACAA′3; ZO-3: 5′ CTGACATGGAGGAGCTGA′3 and 5′ ACTGAACCTGACCGTACAGCTTAGCTTCCCTTCTGACT′3), GAPDH (5′AAGGTCATCCATGACAACTT′3 and 5′ACTGAACCTGACCGTACAGCCATCCACAGTCTTCTG′3) and CK19 (5′AGCCACTACTACACGACCAT′3 and 5′ACTGAACCTGACCGTACATCGATCTGCAGGACAATC′3).

### 2.6. Western Blotting 

Proteins were extracted from cultured cells with RIPA buffer and quantified using a BioRad protein quantitation kit (Bio-Rad Laboratories, Hertfordshire, UK). The samples were treated with 2 × Laemmle sample buffer, boiled for 5 min at 100 °C and then loaded to 12% SDS PAGE gel for electrophoresis. The protein transfer from the gel onto the PVDF membrane that had been preactivated with methanol was subsequently accomplished using a semidry transfer technique. A 10% milk powder solution was utilized for membrane blocking. The blots were incubated with the respective primary antibody to CLDN9 and GAPDH, and then exposed to the secondary antibody that was HRP-conjugated before being visualized with EZ-ECL solution (Geneflow Ltd., Litchfield, UK).

### 2.7. Cellular Response to Chemotherapy Drugs

Breast cancer cells, with or without CLDN9 knockdown, were seeded into 96-well plates and treated with serially diluted drugs before they were incubated in the incubator. The concentrations of the drugs were, respectively, chosen based on their known IC_50_ and previous studies. After 72 h, the cells were fixed with 4% formalin, stained with 0.5% crystal violet and extracted with 10% acetic acid after washing. The absorbance was measured at 595 nm using a spectrophotometer to detect their respective cell densities. The percentage drug toxicity was calculated as follows:

Percentage drug toxicity = [(Absorbance in untreated well − Absorbance in drug treated well)/Absorbance in untreated well] × 100

The scatterplots of percentage toxicity versus drug concentration were plotted, with the best fit curve used to calculate the respective IC_50_ value.

### 2.8. Immunohistochemical Staining of CLDN9 Protein

CLDN9 staining was carried out using a breast cancer tissue microarray BR1503f (US Biomax, Inc., Derwood, MD, USA), which had 75 cases of breast cancer tissues (in total, 128 samples were utilized for the analysis). After dewaxing and rehydration, the tissue microarray was subjected to antigen retrieval followed by thorough washing in PBS. After blocking nonspecific binding with 10% horse serum, the primary anti-CLDN9 antibody was added and incubated overnight at 4 °C (final concentration: 2 ug/mL). Following extensive washing in PBS, the secondary antibody and tertiary reagents were added, with biotinylated secondary antibody first followed by avidin–biotin amplification with a commercial kit (Vectastain Elite Universal ABC kit, Vector Laboratories Ltd., Peterborough, UK). After further washing with PBS, the slides were further incubated with 3,3′-diaminobenzidine (DAB) solution to allow for chromogenic detection. Finally, the tissue sections were counterstained with hematoxylin, washed thoroughly in tap water and then subjected to dehydration through a graded series of ethanol, prior to being cleared in xylene and mounted in DPX mounting solution (Sigma-Aldrich, Dorset, UK). Staining was visualized using a Leica DM1000 LED microscope (Leica Biosystems Ltd., Newcastle, England, UK). Negative controls were prepared by omitting the primary antibody and only using the secondary universal antibody from the Vectastain Elite ABC kit. The staining pattern and intensity were evaluated by two independent researchers, as previously reported [46].

### 2.9. Immunofluorescence (IFC)

Pretreatment was carried out with an 8-well chamber slide with the medium in the incubator overnight. 10 × 10^4^ cells were seeded into each well of the chamber slide. After 36 h of incubation, the medium was discarded, and the cells were fixed with 4% formalin. After washing with PBS, the cells were permeabilized with 0.1% Triton × 100 (diluted with PBS) for 5 min. Nonspecific binding was blocked with 8% horse serum (dilute with PBS) for 2 h, and the slide then incubated with primary antibody at a concentration of 1:100 overnight at 4 °C. The slides were subsequently incubated with secondary antibodies tagged with either fluorescein isothiocyanate (FITC, 1:500) or tetramethylrhodamine isothiocyanate (TRITC, 1:500) (Sigma-Aldrich, Dorset, UK), together with 6-Diamidino-2-phenylindole (DAPI, 1:1000) (Merck Millipore, Watford, UK). The slides were washed and mounted with FluoSave (Calbiochem, Nottingham, England, UK) in preparation for photographing. Images were captured using an Olympus microscope and photographed with a Hamamatsu digital camera.

### 2.10. Patients’ Response to Chemotherapies and Evaluation

We used a comprehensive public database that contains breast cancer patients with their therapeutic options recorded [47]. The database took the approach of ROC (receiver operating characteristic curve), allowing for classification of patients’ sensitivity to a therapy. Here, the AUC (area under the curve) values and the statistical value for sensitivity to treatment were recorded. Additionally, the levels of the respective gene expression of the gene of interest were also displayed together with their statistical power (using a Mann–Whitney U test).

### 2.11. Statistical Methods

Statistical analyses were carried out using SPSS (version 27.0). Groupwise comparisons were conducted using a Kruskal–Wallis test and ANOVA where applicable. Pairwise comparisons were performed using a Mann–Whitney U test, as indicated in the text. Kaplan–Meier method and log rank test were used to run survival analysis. Univariate and multivariate analyses were conducted using Cox regression model. Classification analysis was achieved using the receiver operating characteristic (ROC) method.

## 3. Results

### 3.1. Transcript Levels of CLDN9 in Breast Cancer Tissues

Breast cancer tissues expressed significantly elevated levels of CLDN9 transcript (*p* = 0.035) (Table 1). Patients who died of breast cancer and who developed breast cancer-related incidence had a raised level of the CLDN9 transcript, although these were marginally statistically significant. It was interesting to note that levels of CLDN9 transcript were significantly correlated with transcripts of ZO-1 and ZO-3, but not ZO-2 (Table 2).

### 3.2. CLDN9 Protein Expression in Mammary Tissues

**Figure 1:**
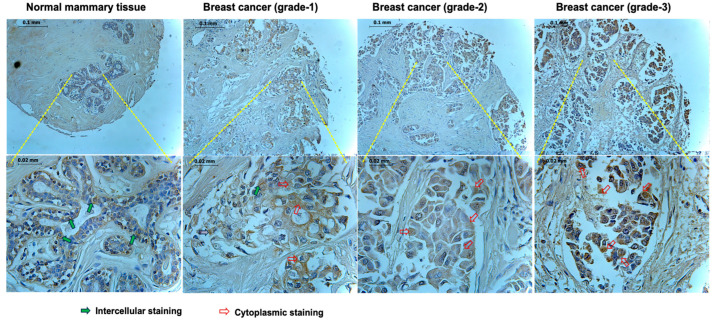
Immunohistochemical detection of the CLDN9 protein in normal mammary tissue (left panel) and breast cancer tissues of different grade (right three panels). In normal tissues, CLDN9 was seen in the residual mammary epithelial cells at the apical regions (green arrows) and to a degree in cytoplasmic regions (open red arrows). In breast cancer, however, the staining was seen in a diffused pattern and was primarily in the cytoplasmic region of the cells (open red arrows).

We evaluated the protein distribution in representative samples of mammary tissues and breast cancer tissues (Figure 1 and Table 3). As shown in Figure 1, normal mammary tissues indicated the presence of CLDN9 protein in areas representing tight junctions (Figure 1, arrows in green). In tumor tissues, the staining appeared to be more diffuse and seem in cytoplasmic regions rather than in the junctional regions (Figure 1, open red arrows), a pattern similar to that found with the ZO family proteins, as previous reported [48]. The analysis of the CLDN9 staining (Table 3) also indicated the pattern changing between normal tissues and tumor tissues.

### 3.3. CLDN9 and Patient Clinical Outcome

**Figure 2:**
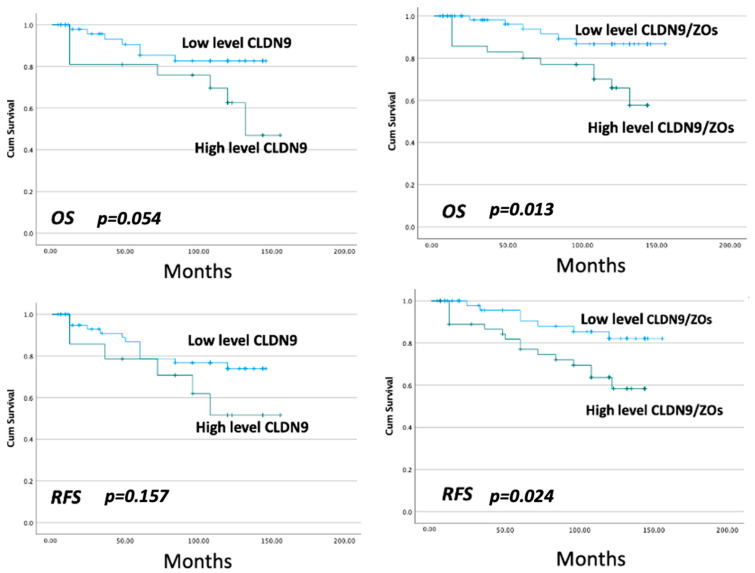
CLDN9 (**left panel**) and integrated expression of CLDN9/ZO-1/ZO-3 (**right panel**) in evaluating the overall survival (OS) and relapse-free survival (RFS) using Kaplan–Meier survival analysis.

Patients with high levels of CLDN9 had a shorter, yet statistically nonsignificant overall survival (OS) than those with low levels (*p* = 0.054) (Figure 2). A weaker link was seen with relapse-free survival (RFS) (*p* = 0.157). Owing to the nature that ZO proteins are critical anchorage proteins for CLDN9 in the cells and that CLDN9 was significantly correlated with ZO-1 and ZO-3, we further integrated the expression pattern of CLDN9, ZO-1 and ZO-3 and analyzed against patient outcome. As can be seen in Figure 2, the expression signature of integrated CLDN9, ZO-1 and ZO-3 had a marked value in significantly predicting both OS (*p* = 0.013, hazard ratio (HR) = 0.1472) and RFS (*p* = 0.024, HR = 1.1148). Integrated expression also showed a high significant independent value in multivariate analysis (Table 4). The CLDN9/ZO expression does provide an excellent prediction value for OS (*p* = 0.004) and for RFS (*p* = 0.010).

### 3.4. CLDN9 and Hormone Status Outcome

**Figure 3:**
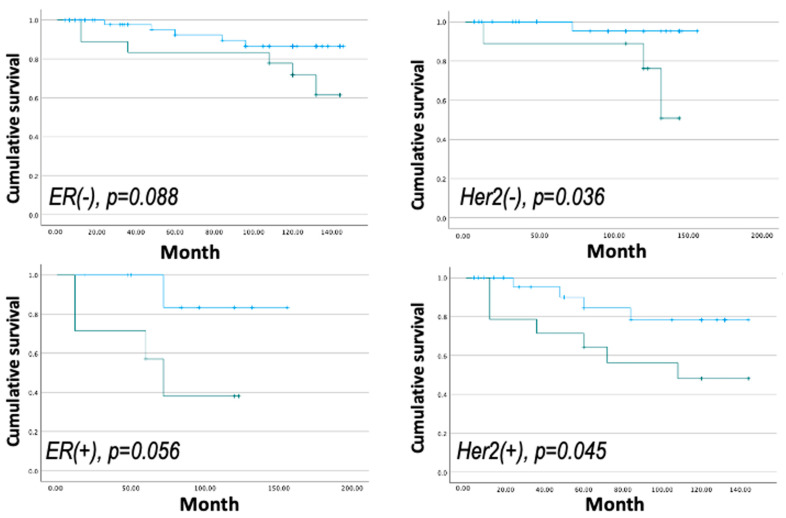
CLDN9/ZO expression signature and the overall survival (OS) in subgroups of patients with different receptor status.

CLDN9 was found to be expressed at different levels depending on receptor status (Figure 3). This inspired us to further investigate if the expression signature had value in the subgroups with different receptor status. As shown in Figure 3, analysis in subgroups did not markedly improve the prediction in overall survival in subgroups with different hormone receptor status, suggesting that this signature may operate well irrespective of the receptor status of breast cancer.

### 3.5. CLDN9 and Patient Response to Drug Treatment

From previous reports that claudins are important markers in predicting patient response to therapies, we conducted an analysis on the available TCGA dataset (accessed via ROCplot.com) and explored the possible link between CLDN9 and patient response to drugs.

#### 3.5.1. High Expression of CLDN9 and Patient Resistance to Chemotherapies

**Figure 4:**
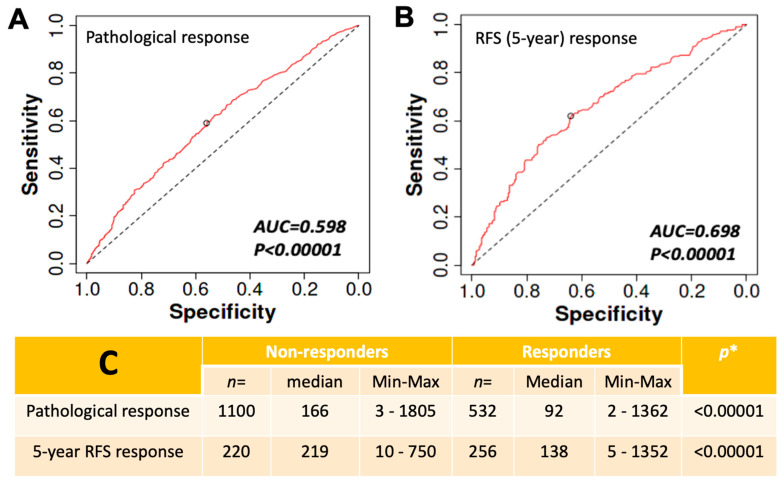
High levels of CLDN9 transcript are linked to patient drug resistance. (**A**,**B**): ROC analysis of patient pathological (**A**) and 5-year relapse-free survival (RFS) (**B**) to chemotherapies. There is a significant connection between CLDN9 transcript and patients’ responses. (**C**) CLDN9 transcript levels in the respective group. Patients who resisted (non-responders) chemotherapies had significantly higher levels of CLDN9 compared with those who responded to treatment in both pathological assessment and RFS assessment. Data from the TCGA dataset (ROCplot.com, accessed on 23 June 2023). * by Mann-Whitney U test.

CLDN9 transcript levels have a significant impact on breast cancers and patient responses. Shown in Figure 4A,B are analyses based on the ROC model in the pathological response (A) and 5-year RFS response (B). It was clear that by both assessment methods, CLDN9 is highly significant in distinguishing patients’ responses. When the levels of CLDN9 were analyzed in the responders (sensitive) and non-responders (resisted) to chemotherapies, the non-responders (resisted) had significantly higher levels of CLDN9 transcript than the responders (Figure 4C).

#### 3.5.2. Expression of CLDN9 and Patient Resistance to Chemotherapies in Relation to Hormone Receptor Status and Molecular Subtypes

**Figure 5:**
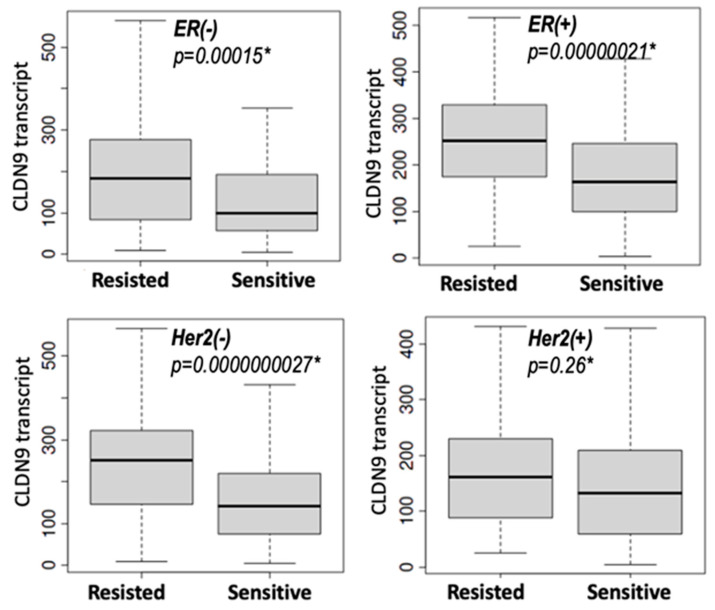
CLDN9 transcript expression and patient response to chemotherapies in relation to hormone receptor status (5-year RFS survival) (data from the TCGA dataset ROCplot.com, accessed 23 June 2023). High levels of CLDN9 transcript in ER-positive and negative both linked to resistance (**top panel**), as well as Her-2 negative tumors (**bottom left**). However, Her-2 positive tumors had little connection with drug responses (all drugs). * Mann–Whitney U test. Shown are median and the interquartile range of the CLDN9 transcript.

When receptor status was considered, it was found that patient response to chemotherapies in ER positive and ER negative tumors showed the same trend that patients who resisted chemotherapies had significantly high levels of CLDN9 transcript compared to the sensitive group (*p* < 0.001 in both groups) (Figure 5 and Table 5). The same link between CLDN9 expression and drug resistance was seen in Her-2-negative tumors (*p* = 0.0000000027). However, it was noteworthy that in Her-2-positive tumors, there was no significant difference between responders and non-responders, leading us to investigate further using in vitro methods (Figure 5 and Table 5).

We subsequently explored the relationship between CLDN9 expression in molecular subtypes. As shown in Table 5, high levels in tumors where Her-2 was negative are linked to resistance. This includes ER(+)/Her-2(−) tumors (*p* = 0.000000009), Luminal-A (*p* = 0.0068) and Luminal-B (*p* = 0.00005) tumors where both are Her-2-negative. What is more interesting is that in triple-negative breast cancers that are both ER- and Her-2 negative, this connection was also highly significant (*p* = 0.000075). Conversely, in the two subgroups that were Her-2 positive, namely ER(+)/Her-2(+) and ER(−)/Her-2(+), the relationship was not significant (*p* = 0.14 and *p* = 0.13, respectively) (Table 5). Collectively, the clinical information indicates that the presence of Her-2 in breast cancer decreases the responsiveness of the cells to chemotherapies.

#### 3.5.3. CLDN9 Is Not Connected to Endocrine or Anti-Her-2 Therapies

It is very interesting to note that levels of CLDN9 do not appear to be linked to patients’ response to endocrine therapies (AUC = 0.503, *p* = 0.45, by 5-year RFS), or anti-Her-2 therapies (AUC = 0.513, *p* = 0.44, by 5-ear RFS).

### 3.6. Creation of CLDN9 Knockdown Breast Cancer Cell Model

**Figure 6:**
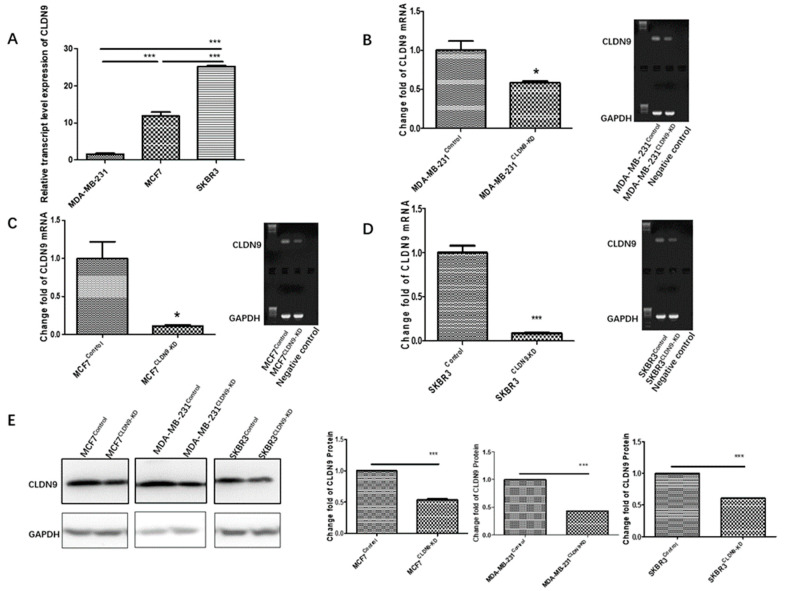
Creation of cell models with reduced expression of CLDN9. (**A**): The baseline expression levels of CLDN9 in the respective cell lines as shown with quantitative PCR (**B**–**D**): The CLDN9 transcript expression in in the respective cell line controls and CLDN9 knockdown cells as confirmed using PCR (right panel) and quantitative PCR (left panel). (**E**): Knockdown of CLDN9 protein in the respective cell lines as shown using protein blotting. * *p* < 0.05, *** *p* < 0.001 vs. control cells.

To corroborate the clinical findings, we established cell models of breast cancer cells with CLDN9 knockdown, based on analyzing the baseline expression levels of CLDN9 in three breast cancer cell lines. The breast cancer cell lines MDA-MB-231 (HER-2−/ER−/PR−), MCF7 (HER-2−/ER+/PR+) and SKBR3 (HER-2+/ER−/PR−), representing the main molecular subtypes of breast cancers as portraited earlier, were employed to generate the CLDN9 knockdown cell models. As shown in Figure 6, the CLDN9 expression in each cell line exhibited a significant reduction following transfections with anti-CLDN9 siRNA, as evidenced with PCR, qPCR and Western blotting analyses.

### 3.7. CLDN9 Expression and Cell’s Response to Chemodrugs

**Figure 7:**
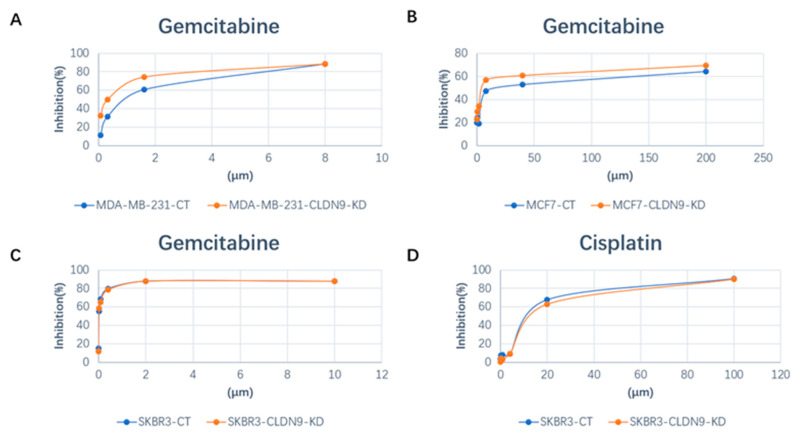
Implication of CLDN9 on breast cells’ response to chemotherapeutic agents over broad concentration ranges. (**A**) MDA-MB-231 cells (gemcitabine 8 μM); (**B**) MCF7 cells (gemcitabine 200 μM); (**C**,**D**): SKBR3 cells (gemcitabine 10 μM and cisplatin 100 μM). CT: control; KD: knockdown.

The aim of this part of the study was to ascertain the correlation between CLDN9 expression levels and chemoresistance across various subtypes of breast cancer cells. We evaluated the IC_50_ values of methotrexate, docetaxel, cisplatin and gemcitabine in the three representative cell lines, encompassing both normal CLDN9 expression levels and those with CLDN9 knockdown. Figure 7 illustrates examples of drug responses within normal CLDN9-expressing cells and CLDN9 knockdown in the breast cancer cell models. Utilizing the cell models established in this study, we validated the responsiveness of these cell lines to chemotherapy agents. As shown in Table 6, knockdown CLDN9 in the Her-2-negative MCF7 and MDA-MB-231 cells sensitized the cells in their response to gemcitabine, docetaxel, methotrexate and, to some degree, cisplatin. However, in the Her-2(+) SKBR3 cells, knockdown CLDN9 only had marginal effects on the cells’ response to these drugs (Table 6).

**Figure 8:**
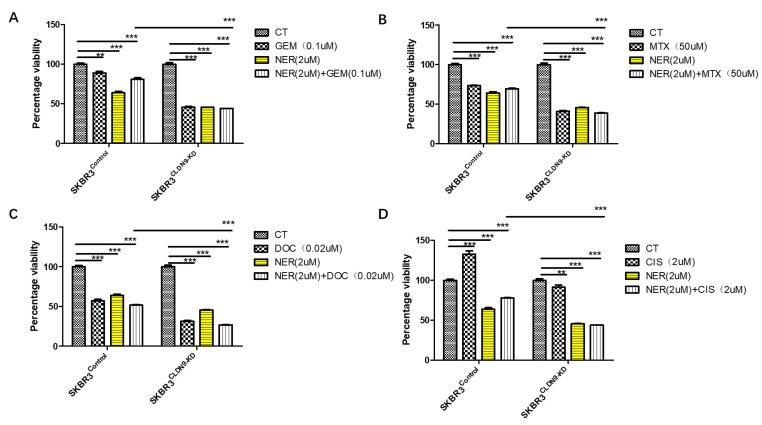
Inhibition of Her-2 and SKBR3 cell’s response to chemotherapeutic drugs in relation with CLDN9 expression. SKBR3 wild-type cells were tested on its wild type (Her-2-positive and CLDN9-positive) and its submodel, CLDN9 knockdown (CLDN9-KD). Neratinib (NER) had a significant inhibitory effects on the growth of both models. However, following blocking of Her-2 with neratinib, the CLDN9-KD SKBR3 cells became more sensitive to Gemcitabine (**A**), Methotrexate (**B**), Docetaxel (**C**), Cisplatin (**D**) (NER plus the respective drugs in SKBR3/CLDN9-KD cells). ** *p* < 0.01, *** *p* < 0.001 vs. control cells.

**Figure 9:**
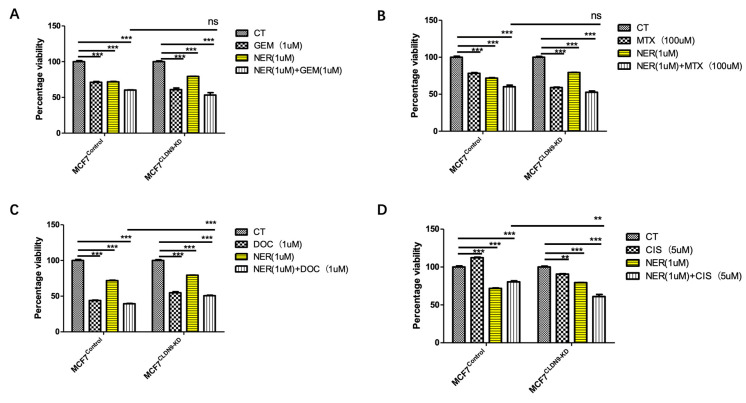
Inhibition of Her-2 and MCF7 cells’ response to chemotherapeutic drugs in relation with CLDN9 expression. MCF7 cells were tested on its wild type (Her-2 negative and CLDN9 positive) and its submodel, CLDN9 knockdown (CLDN9-KD). Neratinib (NER) had some inhibitory effects on the growth of both models. However, blocking Her-2 with neratinib did not influence cells response to Gemcitabine (**A**), Methotrexate (**B**), Docetaxel (**C**), Cisplatin (**D**) in both cell models. ns > 0.05, ** *p* < 0.01, *** *p* < 0.001 vs. control cells.

**Figure 10:**
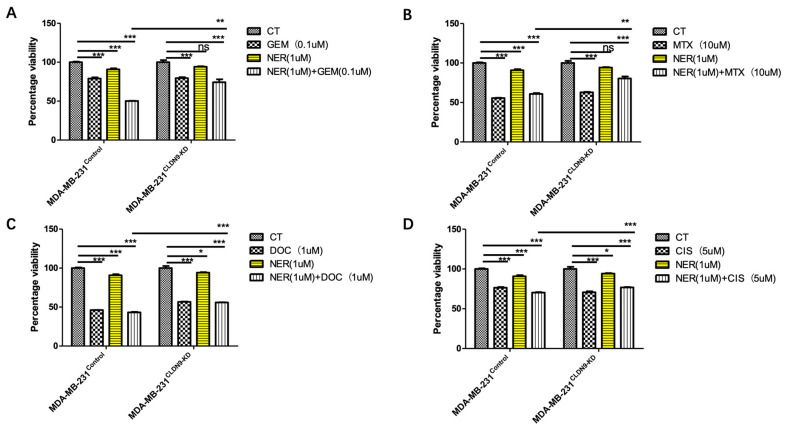
Inhibition of Her-2 and MDA-MB-231 cells’ response to chemotherapeutic drugs in relation with CLDN9 expression. MDA-MB-231 cells were tested on its wild type (Her-2-negative and CLDN9-positive) and its submodel, CLDN9 knockdown (CLDN9-KD). Neratinib (NER) had some inhibitory effects on the growth of both models. However, blocking Her-2 with neratinib did not influence cell responses to Gemcitabine (**A**), Methotrexate (**B**), Docetaxel (**C**), Cisplatin (**D**) in both cell models. ns > 0.05, * *p* < 0.05, ** *p* < 0.01, *** *p* < 0.001 vs. control cells.

As indicated in Table 5, in patients bearing Her-2 breast cancer, CLDN9 appeared to have lost its value in predicting patient response to chemotherapies. In order to test if this connection can be replicated in vitro, we employed a Her-2-positive cell, SKBR3, and applied a permanent Her-2 inhibitor, neratinib, with or without chemotherapeutic drugs. As shown in Figure 8, both neratinib and chemotherapy drugs (except cisplatin) exert cytotoxicity on SKBR3 control cells. However, with CLDN9 knockdown in SKBR3 cells, cells became more sensitive to neratinib and chemodrugs. Additionally, the combination of neratinib and chemodrugs appeared to render more toxicity than using either the Her-2 inhibitor alone or the chemodrug alone. This effect was not seen with MCF7 (Figure 9) nor with MDA-MB-231 (Figure 10) cells, both of which are Her-2-negative in that the control cells and CLDN9kd cells responded similarly to neratinib, chemodrugs and their combinations, as seen in MCF-7 (Figure 9) and MDA MB-231 (Figure 10).

### 3.8. The Relationship between CLDN9 and ZO-1 and ZO-3, Respectively

**Figure 11:**
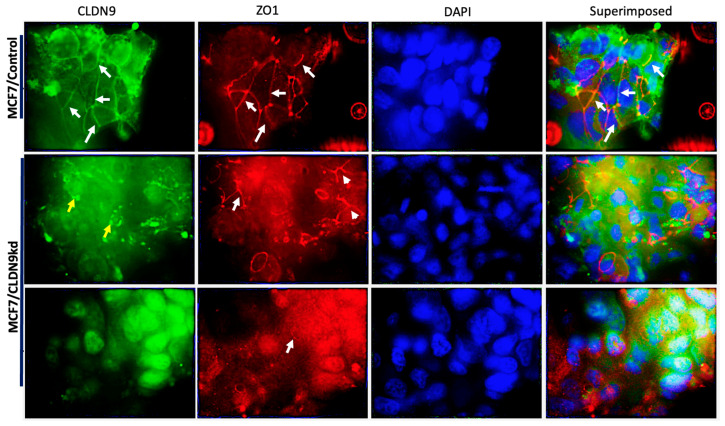
The correlation between CLDN9 and ZO protein in terms of colocalization and expression levels in the MCF-7 cell models. CLDN9 (green) and ZO-1 (red), as shown using IFC in wild-type (top panel) and CLDN9 knockdown (bottom two panels). In control wild type cells, CLDN9 (green) and ZO-1 (red) were seen in the tight junction areas (indicated by white arrows) and had a colocalization pattern (top right imposed image). Following CLDN9 knockdown, there were changes in the localization of ZO-1 in that it partly remained in the tight junctions owing to its interaction with other tight junction proteins (middle panel). In contrast, after losing a majority of the CLDN9 proteins, the remaining CLDN-9 was localized in regions unrelated to tight junction (yellow arrows in the middle panel and bottom panel).

To further ascertain the intrinsic relationship between CLDN9 and ZO-1, we utilized immunofluorescence staining to explore expression and localization of the ZO proteins in CLDN9 knockdowns, MCF-7, MDA-MB-231 and SKBR3 cell models. We found that CLDN9 protein was colocalized with ZO-1 proteins in the control cells (Figure 11, top panel representative images from MCF-7 cells). Following CLDN9 knockdown, most cells showed reduced levels of CLDN9 protein staining. In the CLDN9 knockdown cells, ZO-1 either remained in the tight junction areas (Figure 11 middle panel) or relocated to regions beyond tight junctions (Figure 11, middle and bottom panels) These data preliminarily demonstrated a correlation and colocalization between CLDN9 and ZO-1 protein in control cells that had expression of CLDN9 protein. When CLDN9 is lost or reduced, ZO-1 localization to tight junctions is weakened, owing to its interaction with other tight junction proteins.

## 4. Discussion

In this study, we report, for the first time, that claudin-9 (CLDN9) is expressed in mammary tissues and that the increased expression in breast cancer, particularly when co-expressed with its anchorage protein ZO1 and ZO3, forms a significant predictor for the survival of patients. This study further demonstrates that the expression pattern of CLDN9 has an important impact on patient response to chemotherapies, in that high levels of CLDN9 indicated chemoresistance, except in Her-2-positive tumors in which CLDN9’s predictive power to drug response is lost. Using in vitro cell models, we further demonstrated that knockdown of CLDN9 in Her-2-negative breast cancer cells gave rise to cells that were more sensitive to chemotherapy drugs, a finding not seen in Her-2-positive SKBR3 cells. Additionally, blocking Her-2 with a permanent Her-2 inhibitor, neratinib, and CLDN9 knockdown in SKBR3 cells sensitized the cells to drugs, a finding not seen in Her-2-negative MCF7 and MDA-MB-231 cells.

CLDN9 has been known to be a transmembrane protein located in the tight junction area of many cell types. However, its role in cancers has been little studied and certainly not in breast cancer. In a continued effort to establish the role of tight junction and tight junctional proteins in breast cancer, we determined CLDN9 expression in breast cancer. First, we confirmed that both CLDN9 transcript and the CLDN9 protein are seen in human mammary tissues and breast cancer tissues. At transcript levels, breast tissues express significantly high levels and there is a clear indication that high levels of CLDN9 are associated with poor clinical outcome of the patients; however, this connection is not seen with Her-2-positive breast cancers. At the tight junction, CLDN9 is known to be anchored to the cytoskeleton by interacting with the subcoat proteins, Zonula Occludens (ZOs), and together they may play roles in orchestration [49]. Here, we also show that expression of CLDN9 is significantly correlated with ZO-1 and ZO-3 and the integrated co-expression of CLDN-9, ZO-1 and ZO-3 markedly improve the prognostic value in breast cancer. This therefore establishes CLDN9 in combination with ZO-1 and ZO-3 as a significant prognostic indicator for patient survival in breast cancer. Here, our limited data show that CLDN9 can be detected at locations of tight junctions particularly in normal mammary tissues. However, the pattern appears to change in breast cancer tissues, in that more cytoplasmic staining appears to be present. CLDN9 and ZO protein colocalization was partly confirmed in our cell models, in which CLDN9 and ZO-1 proteins were colocalized to the tight junctions in controls cells. However, following CLDN9 knockdown, the ZO-1 localization to the tight junction was partly affected, owing to the fact that it is an anchorage protein for rather large number of tight junction transmembrane proteins. Another intriguing observation was the expression pattern of the CLDN9 mRNA and proteins in relation to tumor grade. There appears to be high levels of *CLDN9* gene transcript in grade 1 tumors, compared with grade 2 and grade 3 tumors (Table 1). Yet, when assessed at protein levels using the immunohistochemical method (on a different cohort), there does not appear to be a significant difference between different grades (Table 3) and instead the difference seems to reside in the cellular location of the proteins, namely membranous versus cytoplasmic. This inconsistency between transcript and protein may be due to the variance in the transcript and relative smaller size of the cohort. Indeed, there does not appear to be a significant difference between different grades in a large TCGA transcript database, suggesting that CLDN9 gene transcript levels may not significantly associated with tumor grade. A larger cohort would be desirable to validate in future studies. Collectively, this needs to be further confirmed, and the membrane and cytoplasmic CLDN9 proteins need further investigation with regard to their possibly different roles.

The other highly interesting finding is the highly significant connection between CLDN9 and drug response in breast cancer. High levels of CLDN9 transcripts clearly indicate resistance to chemotherapies in all breast cancer subtypes, including triple-negative breast cancers (TNBC), other than Her-2-positive breast cancers. Where Her-2-positive is detected, there is no difference in the levels of CLDN9 between those who resisted and those who responded to chemotherapies. This is very interesting and suggests that the presence of Her-2 receptor kinase may offer a yet unidentified mechanism by which it overcomes CLDN9-mediated drug resistance. This possibility is partially confirmed in our experiment in that when Her-2 is inhibited by way of therapeutic Her-2 inhibitor and CLDN9 is reduced by genetic knockdown, we were able to sensitize the otherwise resistant Her-2-positive breast cancer to chemodrugs. Additionally, the Her-2 inhibitor and CLDN9 inhibition may have additive value in sensitizing cancer cells. This finding thus has clinical implications. In Her-2-negative breast cancers including TNBC breast cancer, high CLDN9 expression generally indicates a likelihood of patient resistance to chemotherapy, which may be reflected in the poor clinical outcome of the patients. However, in patients with Her-2-positive breast cancers, if there are concurrently low levels of CLDN9, a combination of Her-2 inhibitor and chemotherapies may offer benefits to the patient; a clinical study would be highly valuable to confirm this.

The cellular mechanism by which CLDN9 confers drug resistance remains to be elucidated. CLDN9 is not alone in this regard. Previous studies have shown that CLDN2, CLDN6 and CLDN7 are involved in patient response to chemotherapies in cancers including breast cancer and colorectal cancer, yet with no clear mechanism identified. CLDN proteins are well known components of tight junctions, the structure and function of which controls apical and paracellular permeability to molecules including certain drugs. One could argue that high levels of CLDNs in endothelial cells may result in tightly controlled paracellular permeability, resistance drugs to penetrate the endothelium, and preventing them reaching cancer cells from the circulation. This may be an important mechanism in clinical settings. Whilst this is a possibility, it is not entirely supported by the present and indeed others’ findings, as all the findings were confirmed directly on cancer cells minus the presence of endothelial cells. There are likely other mechanisms unrelated to tight junction that may operate here, including the possibility of the junctional role for CLDN9 in cancer cells themselves. Tight junctions in cancer may also govern paracellular permeability and hence intratumor drug penetration; thus, CLDN9 proteins at the tight junctions in cancer cells may participate this restriction of intratumor availability in drugs. However, it is worth noting that most small compound drugs, such as the ones tested in the present study, require specific drug transporters on cell membrane, for example, SLC28A1 and SLC29A1 for gemcitabine. This again suggests other possible mechanism(s) here, including possibility of interplay between claudins and membrane drug transporters in cancer cells such as the solute carrier family (SLC). Indeed, it has been shown that CLDN12 can be affected in coordination with SLC9A3, and, in particular, in brain endothelial cells [50,51,52]. There have been indications that certain SLC members, namely SLC22A5, may be regulated by a common subcoat protein with claudins, such as ZO1 [53]. Thus, the shared intracellular regulatory pathway between CLDN proteins and SLC protein may also influence the response. The high correlation between CLDN9 and ZO1/ZO3 and the integrated pattern between CLDN9 and the two ZOs may provide some indirect evidence here. The brief finding that CLDN9 is also seen in the cytoplasmic regions of breast cancer may contribute to this suggestion. However, many tight junction proteins when “switched off” relocate to the cytoplasm and away from the membrane area. Presently, it is unclear what role the cytoplasmic CLDN9 protein performs in cell functions; this will be very interesting to explore in the future.

## 5. Conclusions

CLDN9 is expressed in mammary tissues and in breast cancer tissues. High levels of CLDN9 in breast cancer present as a potentially significant prognostic indicator for patients with breast cancer who are Her-2-negative. CLDN9 expression is also a pivotal factor in assessing patient responses to chemotherapies. Her-2 is a negating factor for the treatment response prediction value of CLDN9 and negating Her-2 and CLDN9 may enhance breast cancer cell response to chemotherapeutic drugs.

## Figures and Tables

**Table 1:** CLDN9 in mammary tissues and breast cancer tissues.

Category	Subgroup	*n*	CLDN9 (Median (Q1–Q3)	*p* Value *
Tissue type	Normal	33	15 (4–764)	0.035
	Tumour	127	77 (4–4342)	
Grade	1	24	1500 (158–21,800)	
	2	43	41 (3–5418)	0.025
	3	58	31 (4–1442)	0.0036
TNM staging	1	70	125 (6–6170)	
	2	40	57 (3–1467)	0.18
	3	7	226 (32–32,600)	0.59
	4	4	89 (20–5529)	0.76
Clinical outcome	Disease free	90	80 (7–6505)	
	Died of BrCa	16	729 (11–16,375)	0.071
	All BrCa Incidence	28	125 (4–1467)	0.05
ER status	Negative	75	32 (4–700)	0.09
	Positive	38	924 (4–18,520)	
Her2	Her2(−)	57	81 (3–5165)	0.75
	Her2(+)	55	75 (7–1970)	

* Mann–Whitney U test.

**Table 2:** Correlation levels of the CLDN9 transcripts with that of ZO-1, ZO-2 and ZO-3 (Spearman ranked method).

	Spearman’s Correlation with CLDN9	
ZO-1	Correlation Coefficient	0.297 **
	Significance (2-tailed)	0.001
ZO-2	Correlation Coefficient	−0.084
	Significance (2-tailed)	0.374
ZO-3	Correlation Coefficient	0.252 *
	Significance (2-tailed)	0.011

* indicating significance *p* < 0.05; ** indicating significance *p* < 0.001.

**Table 3:** Analysis of the CLDN9 staining in the breast cancer TMA (BR1503f).

	Intensity							
	Negative to Weak (0–1)	Moderate to Strong (2–3)	Membrane		Nucleus		Statistical Significance	
			Positive	Negative	Positive	Negative	Chi Value	*p* Value
Normal (*n* = 3)	0	3	3	0	0	3		
Tumor (*n* = 128)	74	54	19	109	23	105	19.85	0.0013
Grade1 (*n* = 4)	2	2	1	3	2	2		
Grade2 (*n* = 57)	29	28	8	49	13	44	1.849	0.8696
Grade3 (*n* = 28)	16	12	2	26	2	26	7.264	0.2018
T1 (*n* = 4)	4	0	0	4	0	4		
T2 (*n* = 69)	35	34	11	58	11	58	5.191	0.393
T3 (*n* = 25)	15	10	5	20	7	18	4.885	0.4301
T4 (*n* = 15)	9	6	1	14	2	13	3.216	0.6667
HER-2- (*n* = 81)	52	29	12	69	13	68		
HER-2+ (*n* = 3)	1	2	0	3	0	3	2.272	0.8104
HER2++ (*n* = 9)	3	6	2	7	0	9	5.273	0.3834
HER2+++ (*n* = 29)	15	14	5	24	7	22	2.431	0.7869
ER- (*n* = 59)	33	26	10	49	13	46		
ER+ (*n* = 18)	10	8	3	15	1	17	2.519	0.7736
ER++ (*n* = 20)	12	8	4	16	1	19	3.168	0.674
ER+++ (*n* = 25)	16	9	2	23	5	20	1.662	0.8937

**Table 4:** Multivariate analysis of the CLDN9/ZO expression signature against clinical outcome.

Factors	OS		RFS	
	Hazard Ratio	*p* Value *	Hazard Ratio	*p* Value *
CLDN9/ZO signature	2.033	0.004	1.239	0.010
NPI **	3.028	0.089	2.068	0.045
Grade	1.287	0.432	1.275	0.530
TNM staging	1.034	0.902	1.412	0.033
ER status	2.022	0.266	3.896	0.008
Her-2 status	3.016	0.083	7.697	0.008

* Cox regression method. ** NPI: Nottingham Prognostic Index.

**Table 5:** CLDN9 expression in breast tumors with different receptor statuses and with different molecular subgroups. Data derived from ROCplot (accessed on 23 June 2023).

5-Year RFS Response		Non-Responders			Responders			*p* *
		*n*	Median	Min–Max	*n*	Median	Min–Max	
ER status	ER(−)	111	182	10–564	115	99	6–922	0.000034
	ER(+)	109	252	26–750	141	163	6–1315	5.9 × 10^−9^
Her2 status	Her2 (−)	173	250	10–750	183	141	6–1362	7.1 × 10^−11^
	Her2 (+)	47	162	25–432	73	133	5–622	0.12
ER(+)/Her2(−)		88	262	26–750	103	163	12–1352	0.000000009
ER(+)/Her2(+)		21	200	75–432	38	168	5–429	0.14
ER(−)/Her2(+)		26	140	25–343	35	122	7–622	0.13
TNBC		84	218	10–564	80	96	6–439	0.000075
Luminal-A		20	202	59–304	58	126	15–501	0.0068
Luminal-B		90	262	26–750	83	199	5–1352	0.00005

* By Mann-Whitney U test.

**Table 6:** IC_50_ of chemotherapy drugs in breast cancer cell lines with modified CLDN9 expression.

	Gemcitabine (µM)	Docetaxel (µM)	Cisplatin (µM)	Methotrexate (µM)
MDA-MB-231^Control^	0.820	54.612	12.520	107.927
MDA-MB-231^CLDN9-KD^	0.287	31.134	4.723	2.7834
MCF7^Control^	27.261	6.575	13.462	2179.402
MCF7^CLDN9-KD^	7.334	17.876	12.414	1510.961
SKBR3^Control^	0.028	0.100	10.162	1012.070
SKBR3^CLDN9-KD^	0.031	0.086	12.493	706.855
